# THERAPEUTIC ASSESSMENT OF *N*-FORMYL-METHIONYL-LEUCYL-PHENYLALANINE (fMLP) IN REDUCING PERIPROSTHETIC JOINT INFECTION

**DOI:** 10.22203/eCM.v042a09

**Published:** 2021-08-26

**Authors:** J.L. Hamilton, M.F. Mohamed, B.R. Witt, M.A. Wimmer, S.H. Shafikhani

**Affiliations:** 1 Department of Orthopedic Surgery, Rush University Medical Center, Chicago, IL 60612-3806, USA; 2 Department of Internal Medicine, Rush University Medical Center, Chicago, IL 60612-3806, USA; 3 Microbial Pathogens and Immunity, Rush University Medical Center, Chicago, IL 60612-3806, USA; 4 Cancer Center, Rush University Medical Center, Chicago, IL 60612-3806, USA

**Keywords:** Periprosthetic joint infection, surgical site infection, *Staphylococcus aureus*, neutrophil, immunomodulator, *N*-formyl-methionyl-leucyl-phenylalanine

## Abstract

Despite many preventive measures, including prophylactic antibiotics, periprosthetic joint infection (PJI) remains a devastating complication following arthroplasty, leading to pain, suffering, morbidity and substantial economic burden. Humans have a powerful innate immune system that can effectively control infections, if alerted quickly. Unfortunately, pathogens use many mechanisms to dampen innate immune responses. The study hypothesis was that immunomodulators that can jumpstart and direct innate immune responses (particularly neutrophils) at the surgical site of implant placement would boost immune responses and reduce PJI, even in the absence of antibiotics.

To test this hypothesis, *N*-formyl-methionyl-leucyl-phenylalanine (fMLP) (a potent chemoattractant for phagocytic leukocytes including neutrophils) was used in a mouse model of PJI with *Staphylococcus aureus* (*S. aureus*). Mice receiving intramedullary femoral implants were divided into three groups: i) implant alone; ii) implant + *S. aureus*; iii) implant + fMLP + *S. aureus.*

fMLP treatment reduced *S. aureus* infection levels by ~ 2-Log orders at day 3. Moreover, fMLP therapy reduced infection-induced peri-implant periosteal reaction, focal cortical loss and areas of inflammatory infiltrate in mice distal femora at day 10. Finally, fMLP treatment reduced pain behaviour and increased weight-bearing at the implant leg in infected mice at day 10.

Data indicated that fMLP therapy is a promising novel approach for reducing PJI, if administered locally at surgical sites. Future work will be toward further enhancement and optimisation of an fMLP-based therapeutic approach through combination with antibiotics and/or implant coating with fMLP.

## Introduction

Approximately 1-2 % of patients with primary knee and hip replacement and about 3-6 % of patients with revision knee and hip replacement develop a PJI (Izakovicova et al., 2019; Parvizi et al., 2017). Estimated risk of PJI can be over 20 % in patients with a combination of demographic, surgical (*e.g.* prior procedure) and/or comorbidity risk factors (*e.g.* diabetes) (Tan et al., 2018). Moreover, PJI cases are projected to rise significantly within the next two decades due to increased number of joint replacements resulting from increased life expectancy and greater expectations of mobility in the elderly (Izakovicova et al., 2019; Kurtz et al., 2007). This alarming scenario occurs despite many prevention strategies that have been implemented to prevent SSI, including surgical hand antisepsis, reduction of foot traffic in and out of the operating room, use of intraoperative skin antiseptic agents, perioperative glycaemic control, appropriate selection of surgical dressings and prophylactic antibiotics (Allegranzi et al., 2016; Anesi et al., 2017; Bratzler et al., 2004; Campbell et al., 2014; Corona and Singer, 2010; Edmiston et al., 2013; Greif et al., 2000; Kamath et al., 2016; Khoshbin et al., 2015; Kroin et al., 2015; Kroin et al., 2016; Lindsay et al., 2011; Panahi et al., 2012; Parvizi et al., 2017; Sewick et al., 2012; Sidhwa and Itani, 2015; Stannard et al., 2012).

Although multiple bacterial pathogens have been associated with PJIs, *S. aureus* is often reported as the most common cause, accounting for approximately 13-44 % of all PJIs (Aggarwal et al., 2014; Manning et al., 2020; Pulido et al., 2008; Tsai et al., 2019). Treatments for PJIs include long courses of antibiotics, debridement of infected tissue and implant removal and replacement (Pelt et al., 2014; Segawa et al., 1999). However, antibiotic use is associated with emergence of antibiotic resistance, gut dysbiosis, increased risk of *Clostridium difficile* infection, organ cytotoxicity and allergic reactions, and the success rates after these treatments are abysmal, often ranging between 50 to 70 % (Becattini et al., 2016; Jakobsson et al., 2010; Korpela et al., 2016; Langdon et al., 2016; Pelt et al., 2014; Sampson et al., 2016; Segawa et al., 1999; Yassour et al., 2016). There can be devastating consequences for patients that acquire a PJI, including amputation, arthrodesis, antibiotic-induced organ cytotoxicity or even death (Pelt et al., 2014; Segawa et al., 1999). In addition, patients with a PJI have also an increased risk of developing additional morbidities, such as deep-vein thrombosis and pulmonary embolism (Lieberman and Hsu, 2005). These dismal outcomes underscore the need for new approaches to reduce and to treat PJIs.

The human innate immune system can recognise invading pathogens as “non-self” and mobilise its plethora of defences to protect the organism against them (Brubaker et al., 2015; Guo et al., 2015; Janeway and Medzhitov, 2002; Kawai and Akira, 2011; Martinon et al., 2009; Schroder and Tschopp, 2010; Takeuchi and Akira, 2010). Recognition of microbial pathogens by PRRs sets in motion multiple signalling cascades that culminate in the production of pro-inflammatory cytokines, which recruit effector innate immune leukocytes, particularly neutrophils; those, in turn, destroy invading pathogens by various direct or indirect mechanisms such as phagocytosis, bursts of ROS, AMP production and NETs (Brinkmann et al., 2004; Dovi et al., 2004). Unfortunately, pathogens have evolved various mechanisms to dampen innate immune responses, blocking production of pro-inflammatory cytokines as well as inducing cell death and blocking proliferation in target host cells (Faure et al., 2014; Hornef et al., 2002; Lai et al., 2009; Mohamed et al., 2021; Shafikhani and Engel, 2006; Shafikhani et al., 2008; Tolle et al., 2015; Wood et al., 2015a; Wood et al., 2015b). The study hypothesis was that local administration of immunomodulators that can accelerate and direct innate immune leukocyte responses (particularly neutrophils) toward the site of the surgically placed implant would enhance the immune responses toward infection and be effective in reducing PJI, even in the absence of prophylactic antibiotics.

To enhance local immune responses at the time of surgery, fMLP (also known as fMLF) was locally administered at the time of implant surgery in an established PJI mouse model (Bernthal et al., 2010; Bernthal et al., 2014; Hegde et al., 2017a; Thompson et al., 2018). fMLP is a naturally occurring formyl peptide. Formyl peptides are released at injured tissues, such as surgical sites, and invading bacterial pathogens (Li et al., 2016; Raoof et al., 2010). fMLP was chosen because it is a potent chemoattractant for neutrophils and other inflammatory leukocytes (Balazovich et al., 1996; Derian et al., 1995; Madianos et al., 2005; Panaro and Mitolo, 1999; Sabroe et al., 2003). In addition, fMLP interaction with FPR chemokine receptors also activates bactericidal functions in neutrophils, such as superoxide and ROS production, phagocytosis and degranulation (Devosse et al., 2009; Dorward et al., 2015; Sengeløv et al., 1994).

## Materials and Methods

### Animal experiments

The study was approval by the Rush University Medical Center Institutional Animal Care and Use Committee (IACUC No: 19-623). All procedures complied strictly with the standards for care and use of animal subjects as stated in the Guide for the Care and Use of Laboratory Animals (Institute of Laboratory Animal Resources, National Academy of Sciences, Bethesda, MD, USA). C57BL/6J mice were obtained from The Jackson Laboratory. These mice were allowed to acclimate to the environment for 1 week prior to performing the experiments.

### Intra-articular injection

To demonstrate that fMLP was capable of mobilising and directing neutrophil response in the knee joint even in the absence of infection and/or injury, 12-week-old C57BL/6J mice received local intra-articular injection of fMLP (Sigma-Aldrich, F3506) or vehicle control at the right-knee joint. Prior to injection, right hindlimbs were shaved and disinfected using 70 % ethanol followed by iodine. fMLP was prepared at a concentration of 1 μg/μL in a solution of 25 % DMSO and 75 % PBS. Vehicle control was a 5 μL solution of 25 % DMSO and 75 % PBS. Mice were anaesthetised using 2 % isoflurane. A total of 5 μL of fMLP solution or vehicle control was injected into the right hindlimb knee joint using a 5 μL Hamilton^®^ syringe (Hamilton, 7634-01) and 27-Gauge needle, as previously described (Nagao et al., 2017). The dosing of 5 μg of fMLP was chosen based on its maximum solubility in vehicle solution and maximum volume of solution that could be injected into the knee-joint space. The syringe needle was applied to the intra-articular space directly through the patellar ligament with the knee flexed at a 90° angle and needle administration perpendicular to the apex of the flexed knee. A total of *n* = 5 mice received fMLP and a total of *n* = 5 mice received vehicle control. Mice were evaluated for local neutrophil response at the knee joint using *in vivo* BLI at 2 h and 6 h post injection. Mice were imaged using BLI under 2 % isoflurane anaesthesia.

### Preparation of bacteria

A bioluminescent *S. aureus* (Xen36 *S. aureus*; PerkinElmer^®^) was used that was derived from parental strain *S. aureus* ATCC 49525 (Wright). This strain has been used and validated in mouse PJI models (Bernthal et al., 2010; Bernthal et al., 2014; Carli et al., 2017; Hegde et al., 2017a). Xen36 *S. aureus* has been genetically modified to express the modified *Photorhabdus luminescens luxABCDE* operon, which encodes a luciferase enzyme, on the native plasmid, enabling this strain to produce a bioluminescent signal. Bacteria were incubated in TSB overnight on the day prior to surgery. On the day of surgery, Xen36 *S. aureus* was diluted in TSB to a spectrophotometer absorbance measurement of 600 nm at an optical density of 0.5 (against a TSB blank). This was equivalent to ~ 1.0 × 10^8^ CFU/mL of *S. aureus*. This solution was further diluted to 5 × 10^5^ CFU/mL in PBS and kept on ice. At the time of surgery, dropwise inoculation of 2 μL of 5 × 10^5^ CFU/mL in PBS at the open knee-joint surgical site using a 5 μL Hamilton^®^ syringe provided 1 × 10^3^ CFU at the knee joint.

### Mouse model of PJI

To show that an fMLP immunomodulator could reduce PJI, an established PJI model with bioluminescent *S. aureus* was used, as described previously (Bernthal et al., 2010; Carli et al., 2017; Hegde et al., 2017a). Briefly, 2 d prior to surgery, the ventral and lateral surface of the right hindlimb of 12-week-old C57BL/6J mice were shaved. To ensure additional removal of hair, a hair-removal spray was used. The day prior to surgery, mice underwent baseline testing for BLI, pain behaviour, weight-bearing and weight. The day prior to surgery, all non-sterilised instruments and implant material were autoclaved in self-sealing autoclave pouches. On the day of surgery, mice were anaesthetised using a mixture of ketamine (90 mg/kg) and xylazine (4.5 mg/kg). The right leg was disinfected using 70 % ethanol followed by iodine. Using aseptic technique and using a dissection microscope (Zeiss, Stemi 508), a skin incision over the right knee was performed followed by a medial parapatellar arthrotomy. Incisions were performed using a Micro Knives sterile scalpel (10315-12, Fine Science Tools, Foster City, CA, USA). To expose the femoral condyles, lateral displacement of the quadriceps patellar complex was performed. The intercondylar notch was located; a 25-Gauge syringe needle attached to a 3 mL syringe was used to penetrate the intercondylar notch and ream the distal intramedullary canal at a distance of approximately 10 mm. An orthopaedic-grade stainless steel K-wire (diameter 0.6 mm; DePuy Synthes) was surgically placed in a retrograde fashion into the intramedullary canal with assistance of a Pin Holder (Fine Sciences Tools, 26018-17). The distal aspect of the K-wire was cut to a length of approximately 11 mm with a wire cutter leaving approximately 1 mm protruding into the joint space.

With the open knee-joint surgical site and implant exposed, in the fMLP group, mice received a local intra-articular dose of 5 μg of fMLP in a 5 μL solution (25 % DMSO and 75 % PBS). This was the fMLP dose previously used for intra-articular injection at the healthy mouse knee-joint to substantially increase local neutrophil activity (discussed in the Results section). In the control groups, mice received 25 % DMSO/75 % PBS solution as vehicle control. A 5 μL Hamilton^®^ syringe was used to drop the solution into the exposed knee-joint space. The solution was left untouched at the knee joint for 5 min to enhance its absorption. This was followed by administration of 1 × 10^3^ CFU *S. aureus* in a 2 μL solution of PBS or PBS control with use of separate 5 μL Hamilton^®^ syringes into the exposed knee-joint space and on top of the implant. Administration of this and/or similar amounts of bacteria into the exposed knee-joint space has been described previously in a mouse PJI model with *S. aureus* (Bernthal et al., 2010; Bernthal et al., 2014; Carli et al., 2017; Hegde et al., 2017b). Then, the exposed knee-joint space was again left untouched for 5 min to enhance absorption of the solution at the knee joint. Following absorption, the quadriceps complex was reduced back to midline. The knee-joint capsule was closed using 6-0 VICRYL sutures (Ethicon). Then, skin was closed using 6-0 PROLENE sutures (Ethicon). Mice were placed on a warming blanket for recovery following surgery. For analgesia, mice received subcutaneous buprenorphine (0.1 mg/kg) every 12 h for a duration of 48 h following surgery. Following surgery, living mice received assessments through BLI at days 1, 3, 5, 7 and 10; and von Frey filament testing, weight-bearing testing and body weight assessment at day 10. Mice were sacrificed at day 10 for μCT assessment and histological analysis. In a parallel study, mice were sacrificed at day 3 for infection assessment by bacteria count of the peri-implant knee-joint tissue and implant, using CFU analysis. In total, 35 mice received femoral implant placement in the PJI model: *n* = 5 implant control, *n* = 15 implant + *S. aureus, n* = 15 implant + fMLP + *S. aureus*. A total of *n* = 6 mice in each of the two infected groups were sacrificed at day 3 for CFU analysis; these mice were assessed through BLI up to day 3. The remaining mice (*n* = 5 implant, *n* = 9 implant + *S. aureus, n* = 9 implant + fMLP + *S. aureus*) were assessed through BLI up to day 10 as well as von Frey filament testing (day 10), weight-bearing (day 10) and body weight measurement (day 10). Then, these mice were sacrificed at day 10 for gross morphological assessment, μCT analysis and histological analysis.

### BLI

IVIS^®^ Lumina II *In Vivo* Imaging System (PerkinElmer^®^) was used to track neutrophil activity at the knee joint as well as for quantification of bacterial abundance at the knee joint. For the assessment of neutrophil response in mice receiving knee injection of fMLP or vehicle control, BLI was performed at baseline prior to injection as well as at 2 h and 6 h post injection. 8 min prior to each imaging time-point, mice received intra-peritoneal injection of 100 mg/kg of luminol (Sigma-Aldrich, A4685) in PBS solution. Luminol is a chemiluminescent compound that produces a bioluminescent signal in the presence of ROS catalysed by MPO in neutrophils and has been used previously as a measure of neutrophil activity in the joints and subcutaneous tissue of mice (Gross et al., 2009; Tseng and Kung, 2013).

**Fig. 1.**
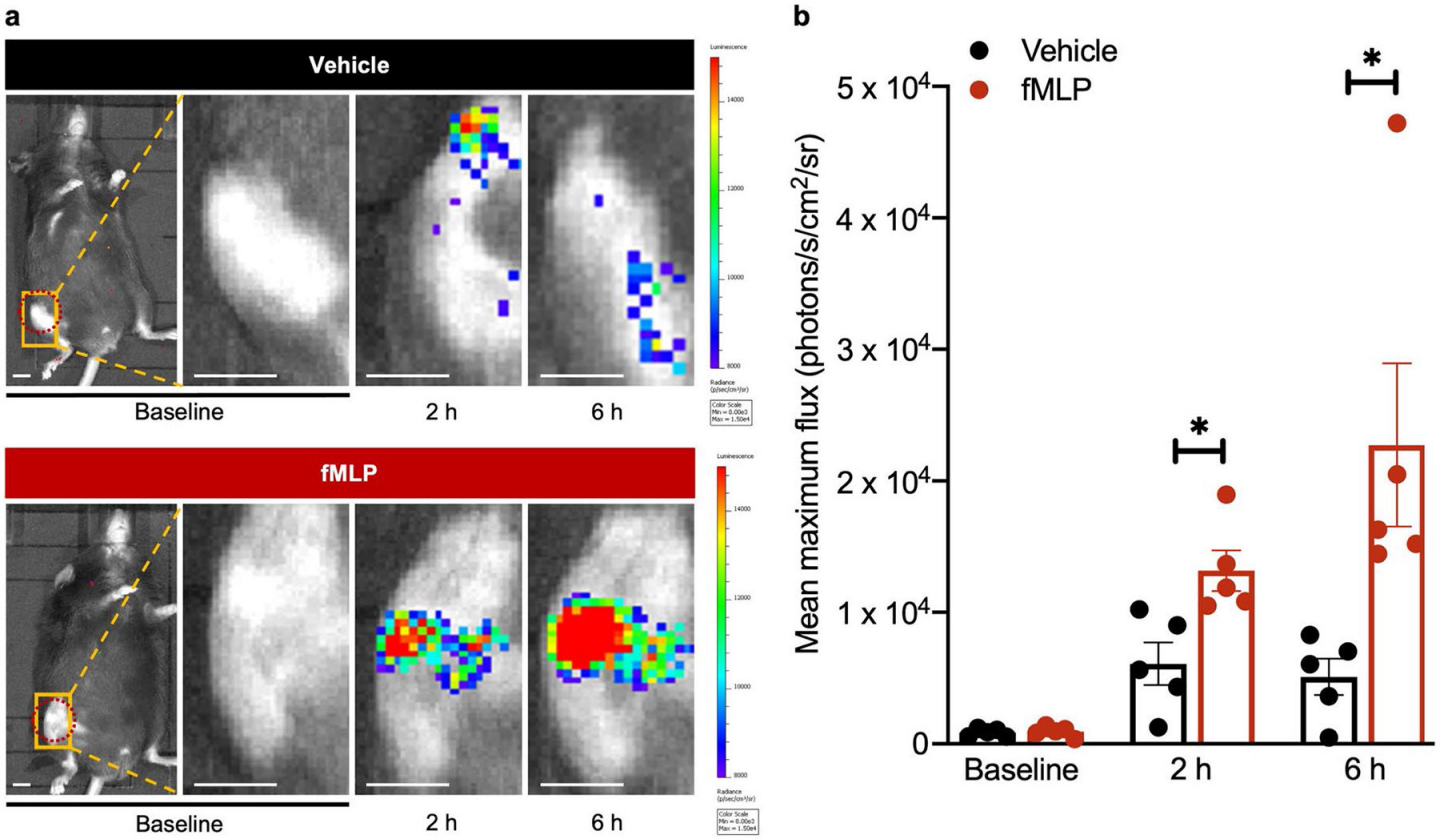
fMLP increased neutrophil activity at the articular joint. (**a**) fMLP (5 μg/5 μL) or vehicle (5 μL PBS + 25 % DMSO) were injected into the right-knee joint. Neutrophil recruitment was assessed by BLI prior to knee injection (baseline) or at 2 h and 6 h post intra-articular knee injection and 8 min post intra-peritoneal luminol injection at each time point. Red-dashed circle represents ROI used for BLI quantification. (**b**) Corresponding tabulated data are shown as mean ± SEM (*n* = 5 mice/group, * *p* < 0.05, Student’s *t*-test). Scale bar: 4 mm.

**Fig. 2.**
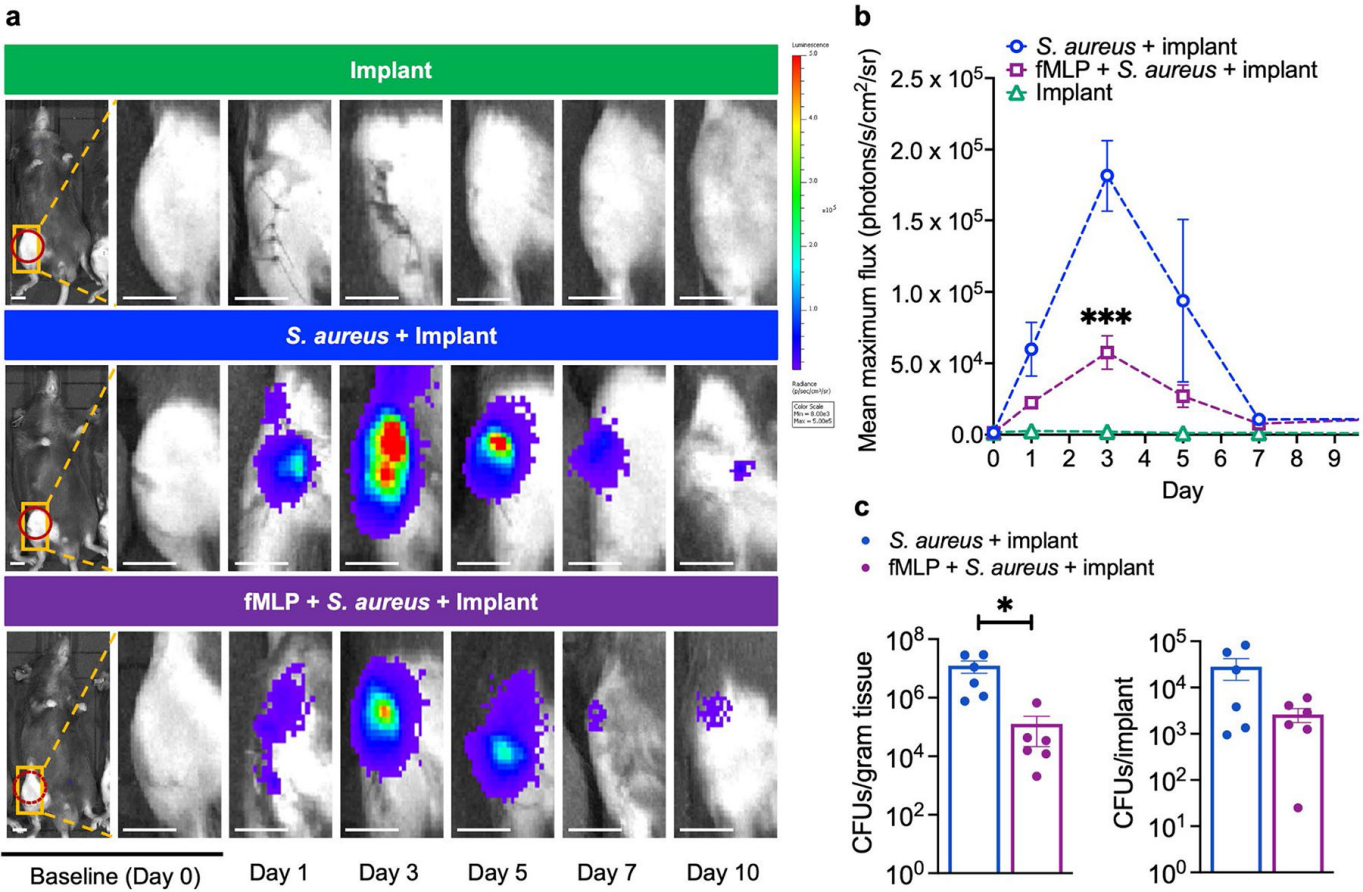
fMLP treatment reduced *S. aureus* infection in a mouse model of PJI. (**a**) Mice received a surgically placed femoral implant and were treated with vehicle alone, 10^3^ Xen36 *S. aureus* or fMLP + Xen36 *S. aureus.* Infection burden was assessed by BLI performed at baseline as well as at days 1, 3, 5, 7 and 10 post-surgery and treatment. Red-dashed circle represents ROI used for BLI quantification. (**b**) Corresponding tabulated data for mean maximum flux (photons/s/cm^2^/sr) at the ROI are shown as mean ± SEM (*n* = 5-15 mice/group, * *p* < 0.05, mixed-effects analysis with *post-hoc* Tukey’s multiple comparison test). (**c**) Quantification of bacterial CFUs at the knee-joint tissue and femoral implant at day 3. Data represented as mean ± SEM (*n* = 6, * *p* < 0.05, Student’s *t*-test). Scale bar: 4 mm.

In the mouse PJI model, BLI to quantify bioluminescent signal from bioluminescent *S. aureus* Xen 36 was performed at baseline (prior to surgery) as well as at days 1, 3, 5, 7 and 10 post-surgery. There is direct correlation between Xen36 *S. aureus* light intensity and quantified tissue and implant bacterial burdened measured by CFU count at the site of the implant and surrounding tissue (Bernthal et al., 2010). In all BLI experiments, exposure time was performed for a total of 5 min. A standard circular ROI with a diameter spanning from the distal 1/4^th^ of the femur and proximal 1/4^th^ of the tibia/fibula was used. The ROI used for both assessment of neutrophil activity and bacterial bioluminescent signal using BLI are represented as red outlines in Fig. 1a and Fig. 2a, respectively. Emission intensity at the ROI over time was quantified using mean maximum flux (photons/s/cm^2^/sr). Mice were imaged using BLI under 2 % isoflurane anaesthesia. A total of *n* = 15 mice in each of the two infected groups were analysed with BLI. For CFU analysis, *n* = 6 mice in each of the infected groups were sacrificed at day 3 and only received BLI up to day 3. The remaining mice (*n* = 5 implant, *n* = 9 implant + *S. aureus, n* = 9 implant + fMLP + *S. aureus)* received BLI assessment up to day 10. BLI images for each group were selected for representation of mean values of mean maximum flux (photons/s/cm^2^/sr) at the ROI.

### Infection burden assessment by CFU analysis at knee joint and implant

A subset of *n* = 6 mice in each of the infected groups (*n* = 6 implant + *S. aureus, n* = 6 implant + fMLP + *S. aureus*) underwent CFU analysis following BLI at day 3 post-surgery. Briefly, mice were sacrificed at day 3 post-infection and the distal 1/4^th^ of the femur and the proximal 1/4^th^ of the tibia/fibula were cut and harvested to isolate the knee joint. Skin was removed from the harvested knee joint and the remaining bone and soft tissue was used for CFU analysis. The implant was removed in an anterograde fashion from the cut end of the femur. Bacterial loads from processed tissue and implants were determined using serial dilution and plating as previously described (Goldufsky et al., 2015; Kroin et al., 2015; Kroin et al., 2012; Kroin et al., 2016; Kroin et al., 2018). Samples were diluted in PBS to produce dilutions ranging from 10^−1^ to 10^−5^ within 96-well microplates. Aliquots of 5 μL were spot-plated at 10^0^-10^−5^ on tryptic soy agar plates and incubated at 37 °C for 24 h. CFU counts were quantified the following day. Bacterial burden was assessed as CFU/g tissue for knee joint and surrounding tissue. Bacterial burden for tissue implant was assessed as CFU/implant.

### Gross morphology analysis

Following sacrifice, mice underwent further imaging with the aid of a Zeiss (Stemi 508) dissection stereomicroscope. Gross morphology was assessed for all mice that were sacrificed at day 10 post-surgery (*n* = 5 implant, *n* = 9 implant + *S. aureus, n* = 9 implant + fMLP + *S. aureus*). Images were acquired following skin incision, to image the knee-joint capsule, and the knee-joint capsule was opened to image the distal femur and implant. Knee capsule width measurements were performed using ImageJ (NIH) using a known reference length from each image. Images of gross morphology for each group were selected as average representatives within each group.

### Histological analysis

The distal aspects of femora and surrounding tissues were fixed in 4 % formaldehyde for 3 d at 4 °C and stored in 70 % ethanol at 4 °C for μCT analysis. Following μCT analysis, tissues were decalcified in 0.5 mol/L EDTA (pH 8.0) for 14 d at 4 °C. Following decalcification, tissues were embedded in paraffin-wax. Serial 5 μm sagittal sections were performed at the distal femur tissues. Sections underwent H&E staining. Sections were imaged using an Olympus (BX43) light microscope. Image J was used to quantify cortical widths and inflammatory areas. Maximum cortical width was evaluated at the distal ventral femur. Maximum cortical width was defined as maximum distance from cortical bone at the ventral surface to the beginning of contiguous marrow space. Furthermore, inflammatory infiltration areas were measured using ImageJ as the largest contiguous areas of inflammatory infiltrates at the ventral 1/3^rd^ of the femur epiphysis. Identification of inflammatory tissue eroding into the bone or marrow space in a mouse PJI model has been previously described (Thompson et al., 2018). Identification of inflammatory infiltrate was based on the following criteria: i) bone destruction, ii) fibrosis, iii) inflammatory infiltrate consisting of leukocytes representing chronic inflammation/osteomyelitis, as previously described (Thompson et al., 2018; Tiemann et al., 2014). Black outline of the ROIs of the ventral 1/3^rd^ of the epiphysis used to measure inflammatory areas are represented in Fig. 4a. Representation of the inflammatory infiltrate areas, used in the calculations, are demarcated by the orange outlines in Fig. 4a. Histological analyses were performed on all mice that were sacrificed at day 10 post-surgery (*n* = 5 implant, *n* = 9 implant + *S. aureus, n* = 9 implant + fMLP + *S. aureus*). Representative histological images for each group were selected based on mean values for maximum cortical width at the distal femora as well as inflammatory infiltration areas.

### μCT assessment

The distal 1/4^th^ of fixed femur tissues at the implant leg at day 10 post-surgery were assessed by μCT (Scanco, μCT50). μCT was performed on all mice that were sacrificed at day 10 post-surgery (*n* = 5 implant, *n* = 9 implant + *S. aureus, n* = 9 implant fMLP + *S. aureus*). 3D as well as mid-coronal μCT sections of the distal femur were evaluated. A maximum width of the distal femur was evaluated using a ventral view 3D μCT image and ImageJ. Increased maximum distal femur width has been previously used as a measure of infection in a mouse PJI model (Thompson et al., 2018). 3D images and mid-coronal μCT sections were used to evaluate the following scoring criteria: i) periosteal reaction; ii) focal cortical loss; iii) trabecular loss; iv) total PJI scores. Total PJI score for each femur was the addition of three scores: i) periosteal reaction; ii) focal cortical loss; iii) trabecular loss. These scores were determined on a scale of 0-2, with 2 being the worst or most severe score. Subjective scoring criteria of bone were developed based on previous clinical radiographic evidence of PJI (Bauer et al., 2006) and mouse PJI model radiographic features and scoring (Carli et al., 2017). Criteria for the following periosteal reaction scores at the distal femur were as follows: 0) no or minimal periosteal reaction restricted to small regions; 1) moderate periosteal reaction with limited changes in cortical surface dimension and congruity; 2) severe and extensive spread of periosteal reaction with moderate to severe changes in cortical surface dimensions and congruity. Criteria for the focal cortical loss scores at the distal femur were as follows: 0) no or minimal areas of focal cortical loss; 1) definitive areas of focal cortical loss found in small regions of the distal femur containing near or full-thickness cortical bone loss; 2) severe focal cortical loss causing full-thickness cortical bone loss spread over large region(s) such as a femoral condyle. Criteria for trabecular loss score at the distal femur were as follows: 0) no or minimal loss of trabecular bone; 1) moderate loss of trabecular bone; 2) severe loss of trabecular bone. Scoring was performed by two blinded observers and an ICC was calculated to estimate inter-rater reliability. ICC values were as follows: i) periosteal reaction, ICC 0.969 (95 % CI 0.926-0.987); ii) focal cortical loss, ICC 0.986 (95 % CI 0.966-0.994); iii) trabecular loss, ICC 0.929 (95 % CI 0.832-0.970); iv) total PJI score, ICC 0.970 (95 % CI 0.928-0.987). Representative μCT images of each group were selected based on mean values for maximum distal femur width as well as scored parameters: periosteal reaction, focal cortical loss and trabecular loss.

### Weight-bearing assessment

In addition to pain, impaired joint function is a common clinical symptomatic feature of PJI (Izakovicova et al., 2019). Weight-bearing at the implant leg was used as a measure of pain and joint function. Mice were assessed for weight-bearing at the right hindlimb at baseline (pre-surgery) and at day 10 post-surgery. Weight-bearing was assessed in all mice that were harvested at day 10 post-surgery (*n* = 5 implant, *n* = 9 implant + *S. aureus, n* = 9 implant + fMLP + *S. aureus*). Mouse weight-bearing was captured using iPhone X (Apple) slow-motion video recording software, as previously described (Carli et al., 2017). Grading at the right hindlimb was as follows: full weight-bearing (3 points); partial weight-bearing (2 points); toe-touch (1 point); non-weight-bearing (0 points). Detailed scoring criteria are illustrated in Video 1 (available on journal website). Scoring was performed by two blinded observers and ICC was 0.972, 95 % CI 0.949-0.984.

### Pain behaviour assessment by von Frey filament testing

One of the most common initial clinical findings or symptoms of a PJI is pain (Izakovicova et al., 2019; Tande and Patel, 2014). To measure pain behaviour in mice, mechanical allodynia was assessed using von Frey filament testing, as previously described (Im et al., 2010; Miller et al., 2012). Mice can demonstrate allodynia, or pain behaviour (demonstrated for instance by leg withdrawal), in response to a normal innocuous stimulus, through application of various levels of mechanical stimulus [von Frey filaments applied at the plantar hind paw (Deuis et al., 2017)]. Mice were placed on top of a metal mesh stand (IITC mesh stand part #408, Woodland Hills, CA, USA) within a small, weighted plastic enclosure. Calibrated von Frey filaments (Stoelting™ Touch Test Sensory Probes, Fisher Scientific) ranging from filament forces of 2.44 g to 4.74 g were used. Filaments were applied to the plantar hind paw with a force requiring the filament to bow. Filaments were held at the plantar surface for 3 s or until a pain withdrawal response was displayed. A modified up-down method was used to calculate the force required to elicit withdrawal of the paw, which was quantified as PWT force (in g). Pain behaviour was assessed in all mice that were sacrificed at day 10 post-surgery (*n* = 5 implant, *n* = 9 implant + *S. aureus, n* = 9 implant + fMLP + *S. aureus*); baseline assessments for all these mice were performed as well.

### Weight

Mice were assessed for body weight at baseline (pre-surgery) and at day 10 post-surgery. Weight measurements were taken to further evaluate a potential response of mice to both surgery and infection, as described previously (Carli et al., 2017), as well as to fMLP treatment. Weight was assessed in all mice that were sacrificed at day 10 post-surgery (*n* = 5 implant, *n* = 9 implant + *S. aureus, n* = 9 implant + fMLP + *S. aureus*). Weight was calculated in g using an electronic scale (Ohaus, Scout SPX, Parsippany, NJ, USA).

### Statistical analysis

Statistical analysis for ICC was performed using SPSS statistics software version 27. The remainder of statistical analyses were performed using Prism software version 8. Comparison between two groups was evaluated using an unpaired Student’s *t*-test. Comparisons between more than two groups were evaluated with one-way ANOVA with *post-hoc* Tukey’s multiple comparison test. Comparisons of more than two groups over time were evaluated with mixed-effects analysis with *post-hoc* Tukey’s multiple comparison test. Data were presented as mean ± SEM. Threshold for significance was set at *p* < 0.05.

## Results

### fMLP treatment increased neutrophil activity in the knee joint

To assess whether fMLP could initiate and direct inflammatory responses toward the implant surgical site, even in the absence of injury and infection, mice received either fMLP or vehicle control in the right knee joint by intra-articular injection. Neutrophil response was assessed by *in vivo* BLI using luminol, which produces a bioluminescent signal in the presence of ROS catalysed by MPO in neutrophils (Gross et al., 2009; Tseng and Kung, 2013). BLI performed at baseline prior to injection demonstrated minimal to no neutrophil activity in the knee joint in either group. As compared to the mock group, mice receiving fMLP exhibited ~ 2-fold higher neutrophil response at the 2 h time point and 3-fold higher neutrophil response at the 6 h time point as assessed by BLI (Fig. 1a,b). These results demonstrated that fMLP was able to mobilise and direct a neutrophil response toward the knee environment even in the absence of infection or surgery.

### fMLP treatment reduced *S. aureus* infection in a mouse model of PJI

To show that an fMLP immunomodulator was able to reduce a PJI, an established PJI model with bioluminescent *S. aureus* was used, as described previously (Bernthal et al., 2010; Carli et al., 2017; Hegde et al., 2017a) and in Materials and Methods. In line with a previous report (Bernthal et al., 2010), bacterial-bioluminescence signal was significantly higher in infected mice as compared to non-infected mice, peaking at day 3 post-infection but declining over time and plateauing at day 7-10 post-infection (Fig. 2a,b). Importantly, infected mice receiving fMLP prior to infection had significantly reduced bacterial-bioluminescence signal at day 3 trending towards reduced signals at days 1 and 5, compared to mice receiving vehicle alone (Fig. 2a,b, *p* < 0.001). Given that the peak infection occurred at day 3 in both mock and fMLP-treated mice, infected implants and tissue surrounding implants were collected and assessed for their bacterial infection burden by CFU analysis, as previously described (Goldufsky et al., 2015; Kroin et al., 2016; Kroin et al., 2018). CFU analyses of the knee-joint tissues at day 3 post-surgery revealed an approximate 2-Log order reduction in *S. aureus* bacterial numbers in the group that received fMLP, as compared to the infected group without fMLP (Fig. 2c, *p* < 0.01). There also appeared to be a trend for CFU reduction on the implant itself in the group that received fMLP, but it did not reach statistical significance. Collectively, these data indicated that an fMLP immunomodulator was able to reduce acute *S. aureus* peak infection in the PJI model.

### fMLP treatment reduced the pathological effects of *S. aureus* infection at the knee joint tissue and bone

**Fig. 3.**
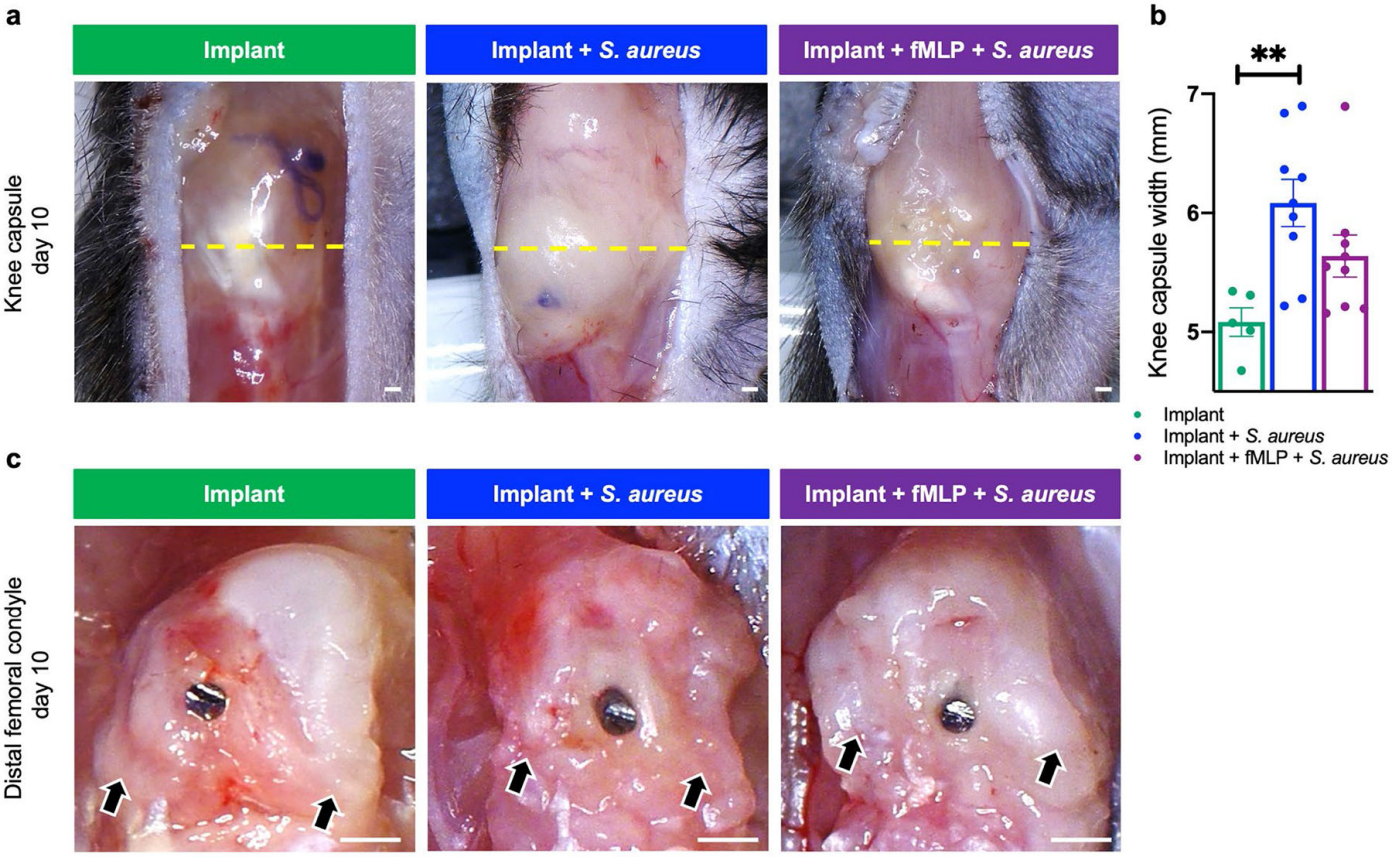
fMLP reduced the pathological effects of infection at the knee-joint tissue surrounding the implant. (**a**) Gross morphological assessment was performed at the knee-joint capsule (knee capsule width in mm/measure of intra-capsular abscess) and distal femur for cartilage/bone erosion surrounding the implant in animals sacrificed at day 10 post-surgery and treatment. Yellow dashed line represents maximum knee-capsule width. Black arrows represent region of the femoral condyles. (**b**) Knee-capsule width plotted as mean ± SEM (*n* = 5-9, ** *p* < 0.05, one-way ANOVA with *post-hoc* Tukey’s multiple comparison test). Scale bar: 0.5 mm.

Bone destruction and joint dysfunction are common clinical signs of PJI (Bozhkova et al., 2020; Izakovicova et al., 2019; Lima et al., 2013). Knee joints and the tissues surrounding the implants were collected at day 10 post-infection and evaluated for the impact of infection with or without fMLP treatment. A striking feature of knee joints in infected mice, particularly in the group without fMLP treatment, was their overall large size as compared to non-infected knee joints (Fig. 3a,b). This increased size was reflective of increased abscess formation within the knee-joint capsule. Infected mice without fMLP treatment had the largest knee-capsule width, which was significantly larger than non-infected mice (Fig. 3a,b, *p* < 0.01). While the fMLP treatment group trended toward having a smaller knee capsule width as compared to mock-treated group, these differences did not reach statistical significance (Fig. 3a,b). Upon opening the knee-joint capsule and removing abscess debris, the distal femur surrounding the implant was evaluated (Fig. 3c). In all groups, there appeared to be some level of adhesive tissue to the distal femur, which was likely a consequence of tissue reaction to implant surgery. In the implant group, characteristic features of the distal femur, such as the femoral condyles were abundantly clear. Infected mice receiving fMLP also appeared to retain somewhat the natural morphology of the distal femur and femoral condyles; however, compared to the non-infected group, there appeared to be more adhesive tissue to the femoral condyles in this group. Mice infected without fMLP treatment had the highest level of adhesive tissue and abnormal morphology of the distal femur of any group. This was most clearly evidenced by a loss of rounded contours of the femoral condyles in the infected group without fMLP, as indicated by black arrows (Fig. 3c, black arrows).

**Fig. 4.**
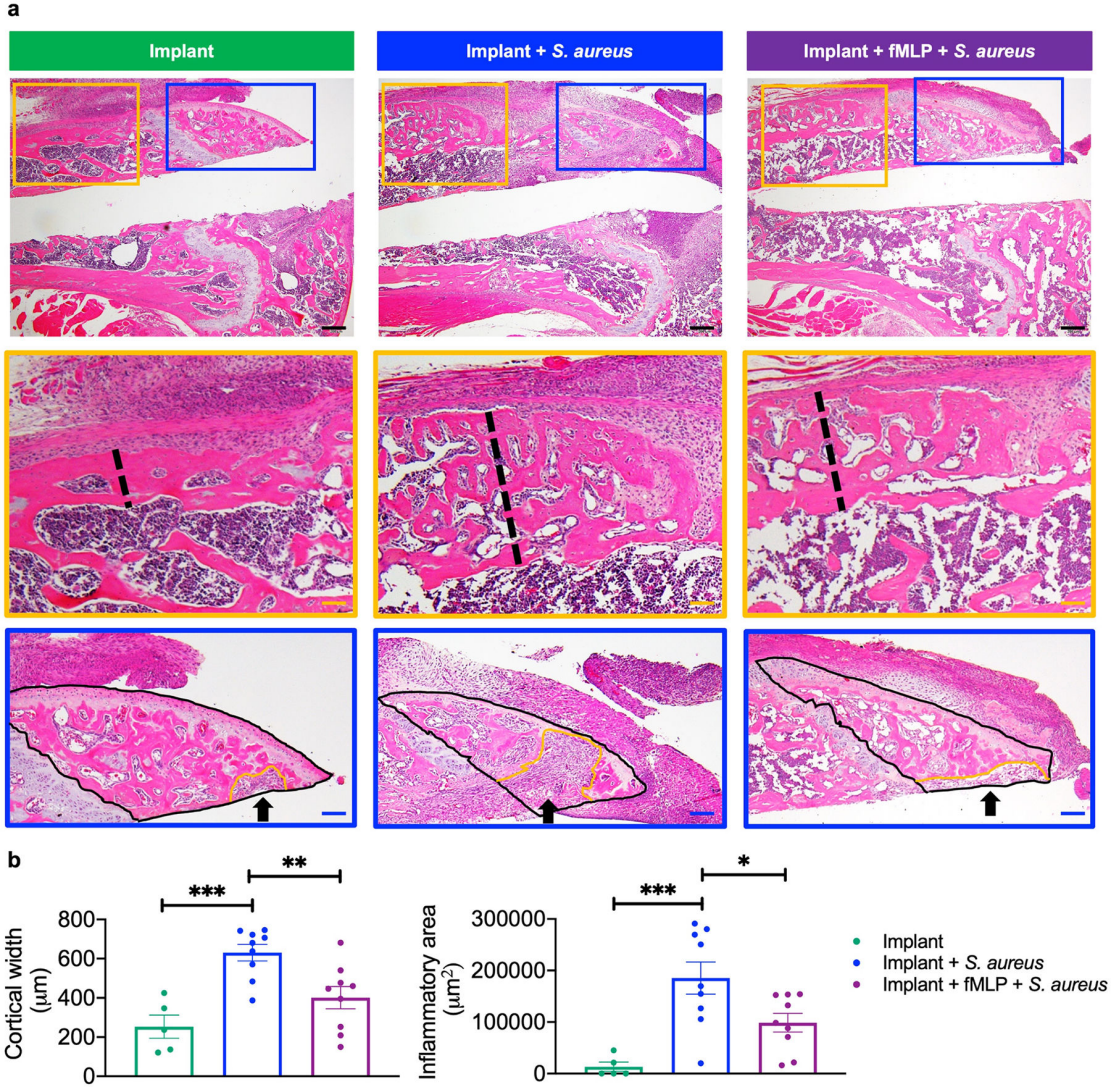
fMLP protected against infection and inflammation-induced bone damage and changes around the implant. (**a**) At day 10 post-surgery and treatment, mice were sacrificed for histological assessment of the distal femur by H&E staining. Two parameters identified in the distal femur were dramatically higher in the infection group without fMLP, maximum cortical width (measure of traditional cortical bone in addition to new adjacent bone formation formed by periosteal reaction, yellow magnification/black dashed line) and inflammatory infiltrate (blue magnification/black arrow). Inflammatory infiltrate was measured as the largest contiguous area of inflammatory infiltrate in the ventral 1/3^rd^ of the femur epiphysis. Within the blue magnification area, ROI for inflammatory infiltrate outlined with black solid line and inflammatory area outlined by an orange line. (**b**) Quantification of maximum cortical width and inflammatory infiltrate. Data represented as mean ± SEM (*n* = 5-9, * *p* < 0.05, ** *p* < 0.01, *** *p* < 0.001, one-way ANOVA with *post-hoc* Tukey’s multiple comparison test). Black scale bar: 200 μm; orange scale bar: 70 μm; blue scale bar: 85 μm.

To gain a better understanding of the effects of infection on the distal femur, with or without fMLP treatment, histological analysis of H&E-stained sections of the distal femur was performed at day 10 post-infection. The fMLP-treated group had significantly smaller cortical bone width as compared to the infected group without fMLP treatment (Fig. 4a,b, indicated by black dotted lines in magnified regions in orange boxes). Increased cortical width was likely a result of stimulation of bone production (periosteal reaction) from the overlying periosteum due to chronic inflammation, as has been reported in the context of infection and inflammation (Rana et al., 2009). The implant group without infection had the smallest cortical width.

Inflammatory infiltrate has been found eroding into the distal femur in a mouse PJI model (Thompson et al., 2018), prompting the assessment of the pathological impact of infection with or without fMLP treatment on inflammatory responses and bone health. Data indicated that within the defined ROI in the ventral 1/3^rd^ of the femur epiphysis, the infected group without fMLP had the largest contiguous area of inflammatory cell and tissue infiltration, which was significantly higher than the fMLP-treated infected group (Fig. 4a,b, *p* < 0.05, black arrow in magnified regions in blue boxes). In contrast, the uninfected implant group had the smallest regions of inflammatory cell and tissue infiltrate, which was likely a result of surgery and implant placement (Fig. 4a,b). Within the ventral 1/3^rd^ of the femur epiphysis, the largest contiguous areas of inflammatory infiltrates were found at the bone-implant interface in all groups.

**Fig. 5.**
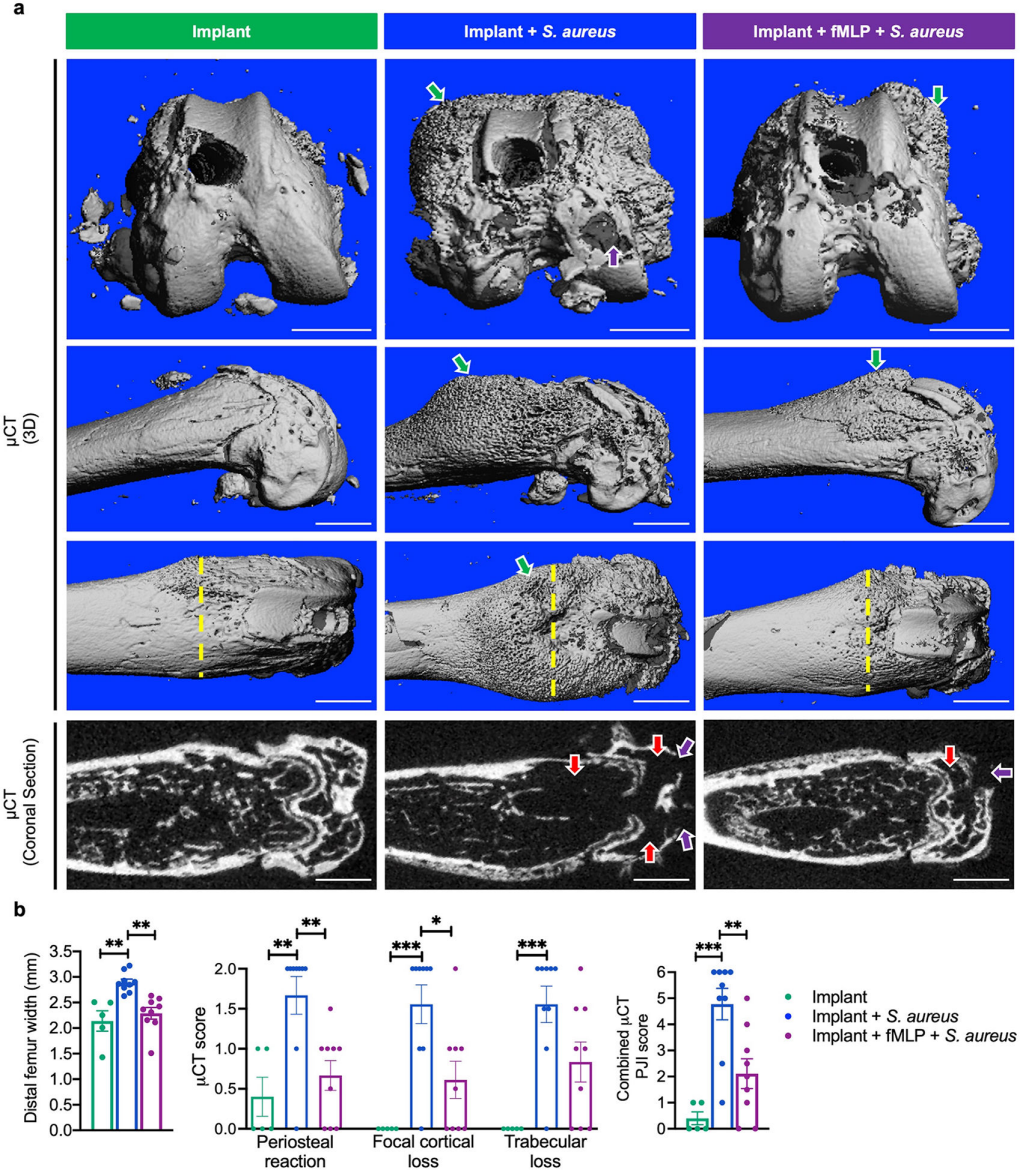
fMLP protected against infection and inflammation-induced bone damage and changes around the implant. Mice were sacrificed at day 10 post-surgery and treatment and femora at the implant sites were evaluated for pathological features as follows. (**a**) Distal femora were assessed by μCT analysis on 3D imaging and mid-coronal sections. Green arrows point to periosteal reaction, purple arrows to focal cortical loss, red arrows to trabecular loss. Yellow-dashed line represents cortical width. (**b**) μCT quantification of maximum femur width in mm (based on ventral-view 3D image), periosteal reaction score, focal cortical-loss score and trabecular-loss score, which were analysed using scoring criteria based on assessment of 3D images and mid-coronal sections by two blinded observers. Scoring criteria were quantified and combined as a single measure called combined μCT PJI score. Data represented as mean ± SEM (*n* = 5-9, * *p* < 0.05, ** *p* < 0.01, *** *p* < 0.001, one-way ANOVA with *post-hoc* Tukey’s multiple comparison test). Scale bar: 1 mm.

The pathological impact of infection with or without fMLP treatment on bone by μCT was assessed. Maximum width of the distal femur was measured on the ventral μCT 3D view. This was in particular used as a measure to quantify periosteal reaction, which would widen the femur width. Similar to the non-infected group, the fMLP-treated group had significantly reduced femoral width as compared to the infected group without fMLP (Fig. 5a,b, *p* < 0.01, indicated by yellow dashed lines). 3D images of the side, dorsal and condyle views, as well as mid-coronal μCT sections were evaluated by two blinded observers and scored for i) periosteal reaction, ii) focal cortical loss, iii) trabecular loss and combination of all three parameters were indexed as a combined μCT PJI score.

As compared to the infected group treated with vehicle, the fMLP-treated group exhibited significantly reduced periosteal reaction (Fig. 5a,b, indicated by green arrows, *p* < 0.01) and focal cortical loss (Fig. 5a,b, indicated by purple arrows, *p* < 0.05). Trabecular loss was significantly higher in the infected group without fMLP, compared to the non-infected group (*p* < 0.001), with a trend towards higher values as compared to the infected group treated with fMLP, but this effect did not reach statistical significance (Fig. 5a,b, indicated by red arrows). When all three parameters were merged into a combined PJI μCT score, it was found that fMLP treatment significantly reduced the combined PJI μCT pathology score as compared to the infected group without fMLP treatment (Fig. 5b, *p* < 0.01).

### fMLP improved behavioural symptoms in a PJI mouse model

**Fig. 6.**
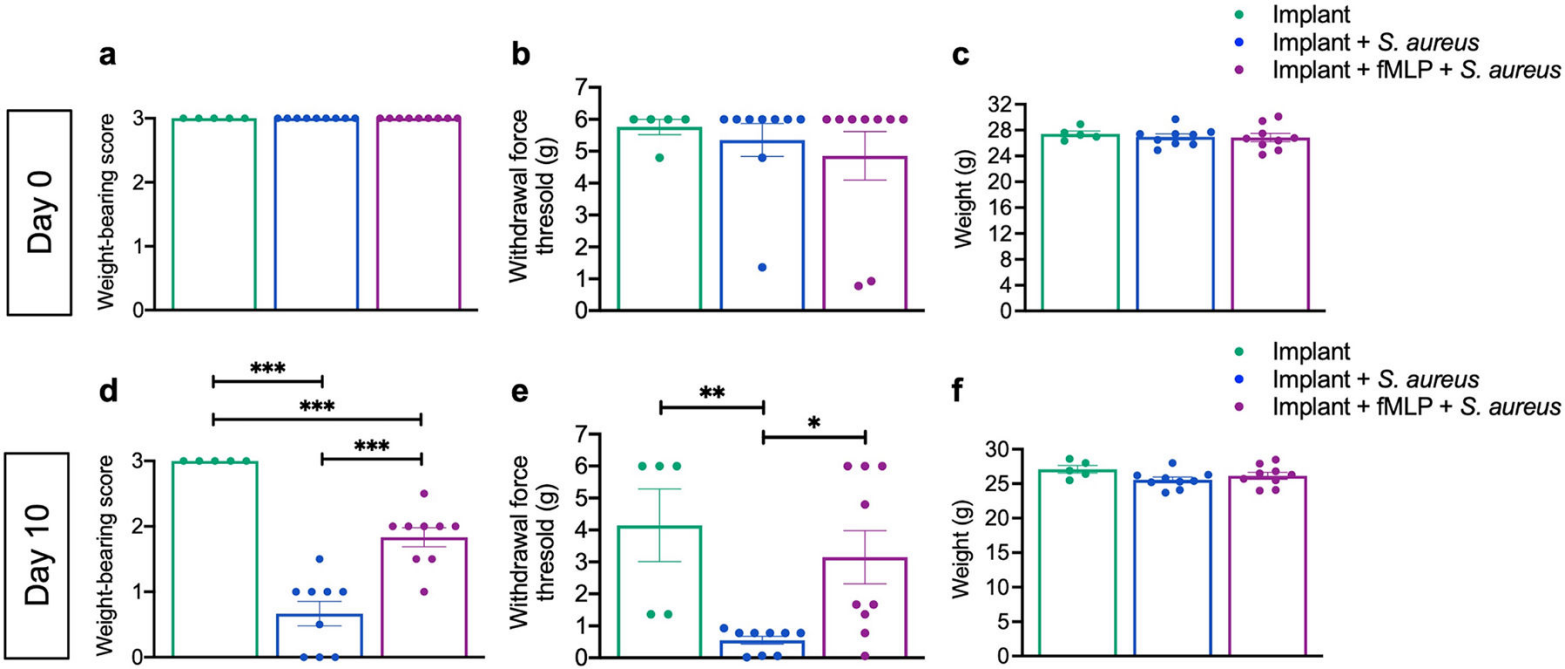
fMLP treatment improved weight-bearing, decreased pain behaviour and had no effect on weight in a mouse model of PJI. Baseline assessment for (**a**) weight-bearing, (**b**) pain behaviour (mechanical allodynia) on von Frey filament testing and (**c**) body weight performed prior to surgery or treatment (day 0). Assessment of (**d**) weight-bearing, (**e**) pain behaviour and (**f**) body weight performed at day 10 post-surgery and treatment. Data represented as the mean ± SEM (*n* = 5-9, * *p* < 0.05, ** *p* < 0.01, *** *p* < 0.001, one-way ANOVA with *post-hoc* Tukey’s multiple comparison test).

Pain and joint dysfunction are common clinical symptom associated with PJI in patients (Bozhkova et al., 2020; Izakovicova et al., 2019; Lima et al., 2013). Pain behaviour was evaluated following two methods: weight-bearing on the right hindlimb (implant leg) and mechanical allodynia with von Frey filament testing, as previously described (Carli et al., 2017; Im et al., 2010; Miller et al., 2012). Weight-bearing was also used as an assessment for joint function (Carli et al., 2017). At baseline and prior to implant placement (day 0), mice in all groups exhibited a similar weight-bearing score and calculated withdrawal force threshold for evidence of mechanical allodynia (Fig. 6a,b). In contrast, at day 10 post-surgery, fMLP-treated infected mice exhibited a significantly improved weight-bearing score on the implant leg as compared to infected mice without fMLP (Fig. 6d, *p* < 0.001). Furthermore, mice receiving fMLP also exhibited significantly improved (increased) withdrawal force threshold as compared to infected mice without fMLP treatment (Fig. 6e, *p* < 0.05). Collectively, these data indicated that fMLP treatment significantly improved the behavioural symptoms that are associated with PJI (Bozhkova et al., 2020; Izakovicova et al., 2019; Lima et al., 2013).

### fMLP treatment did not affect body weight in a mouse PJI model

Prior to surgery, all groups had a similar average body weight (Fig. 6c). At day 10 post-surgery, all groups trended slightly toward having a lower weight; however, this was not statistically significant. Furthermore, at day 10 post-surgery, infected mice without fMLP treatment trended toward a slightly greater weight loss; however, this weight loss was not statistically significant between groups (Fig. 6f).

## Discussion

PJI remains a devastating complication after arthroplasty, leading to pain, suffering, morbidity and a substantial economic burden (Kapadia et al., 2016; Kurtz et al., 2012). There is an urgent need for alternative and/or adjunct measures to antibiotic prophylaxis in addressing PJI. The present study assessed whether fMLP, a potent immunomodulator that recruits and activates inflammatory leukocytes, particularly neutrophils, would be able to control PJI even in the absence of antibiotics. Data indicated that fMLP treatment significantly reduced *S. aureus* infection in an established PJI model. Furthermore, fMLP reduced infection-induced bone and tissue pathologies and, subsequently, infection-induced pain and weight-bearing behaviour in infected animals.

Although infection was significantly reduced in fMLP-treated mice, it was not completely abolished in this model. Whether this was due to low level of fMLP used or the inability of fMLP to engage the adaptive immune responses, which are also needed to fully control *S. aureus* infection (Lin et al., 2009), remains to be investigated. Intriguingly, administration of systemic vancomycin prophylaxis or intra-articular vancomycin powder treatment alone also failed to completely eradicate *S. aureus* PJI in rats, although they were more effective in reducing infection when combined (Edelstein et al., 2017). Since antibiotics and immunomodulators (*e.g.* fMLP) combat infection through different mechanisms of action, combination therapy with both may also be more effective in eliminating a PJI. Future studies are needed to investigate whether fMLP in combination with prophylaxis or topical antibiotics would be more effective in controlling a PJI.

It is encouraging that fMLP treatment reduced infection burden early after surgery and infection, given that bacteria have been shown to form biofilm on the implant surfaces early after infection (Carli et al., 2017; Lamret et al., 2020). Indeed, a high neutrophil to *S. aureus* ratio at the implant surface is a prognostic factor for reduced biofilm production (Ghimire et al., 2019). Bacterial pathogens, including *S. aureus,* are protected from neutrophil killing when embedded in fully mature biofilms (de Vor et al., 2020; Gunther et al., 2009; Kristian et al., 2008). Due to the concern of biofilm formation on the implant surface itself, coating the implant with fMLP might better mobilise neutrophil recruitment directly to the implant interface, thus increasing neutrophil to *S. aureus* ratio at the implant surface to prevent early biofilm formation. Future studies are needed to assess the impact of fMLP therapy (administered intra-articularly or by implant coating; alone or in combination with antibiotics) on biofilm production.

Inflammation and inflammatory responses are proportional to the bacterial burden and infection level and can culminate in severe tissue destruction (Bernthal et al., 2010; Carli et al., 2017). Chronic inflammation in the setting of infection can suppress osteoblast activity as well as enhance osteoclast activity and *S. aureus* infection can directly cause bone destruction, activate osteoclasts and inhibit osteoblasts leading to altered bone remodelling (Wright and Nair, 2010). Substantial evidence of inflammation and bone loss and destruction was found particularly in the infected group without fMLP, as assessed by gross morphology, μCT and histology. Furthermore, in the setting of osteomyelitis, periosteal reaction can occur through subperiosteal spread of inflammation, which in turn elevates and stimulates the periosteum to lay down new layers of bone (Rana et al., 2009). Consistent with this report, significant increases in inflammation and periosteal reaction were found in the mouse PJI model, as assessed by μCT and histological analyses. Importantly, fMLP lowered these infection-induced bone pathologies.

Common clinical signs of PJI include joint pain and joint dysfunction (Bozhkova et al., 2020; Izakovicova et al., 2019; Lima et al., 2013). Intriguingly, infected mice treated with fMLP exhibited significant reduction in pain behaviours and significant improvement in weight-bearing, further highlighting the positive impact of fMLP therapy in reducing PJI. Severe weight loss can be a sign of systemic infection with bacteria such as *S. aureus* (Wu et al., 2017). A slight trend towards weight loss was found in both infected and non-infected groups, although these differences did not reach statistical significance, suggesting that *S. aureus* infection in this model remained local. In line with the present study data, Carli et al. (2017), in a similar PJI *S. aureus* infection study, reported no significant differences in weights between the infected and non-infected groups, although both groups exhibited slightly lower weight at week 1 post-surgery.

There is minimal literature to date on the therapeutic use of fMLP. Interestingly, Shin et al. (2011) evaluated the impact of local treatment with fMLP (delivered in a hyaluronic acid gel carrier) in a rabbit calvaria defect model and demonstrated that fMLP promotes osteogenesis and bone formation at the defect site as compared to vehicle control. Of note, no evidence of increased inflammation was found at the defect site in rabbits treated with fMLP 4 weeks post treatment (Shin et al., 2011), suggesting that fMLP may have a positive effect on bone formation and healing after arthroplasty even in the absence of infection. Further studies should evaluate the positive or adverse impacts of local fMLP therapy on joint and surrounding tissue, as well as animal behaviour, in an uninfected cohort in a PJI model, to further lay the groundwork for its therapeutic use to combat PJI.

Sethi et al. (2015) reported that in rats with an *S. aureus*-contaminated surgically-placed tibial-intramedullary implant, administration of CpG oligodeoxynucleotide, which is found in bacterial DNA and shown to trigger inflammatory responses, led to ~ 67 % reduction in infection burden early after infection but did not prevent the development of chronic infection over time. The present study results were in line with these findings showing that fMLP reduced early infection by a nearly 2-Log order at day 3 but did not completely abolish infection.

## Conclusions

The present proof-of-concept study provided direct evidence in a mouse PJI model that fMLP immunomodulator was effective in reducing acute infection and protecting against infection-induced bone and tissue damage and associated pain. Immunomodulators such as fMLP may provide an alternative or adjunct therapeutic to antibiotics for reducing and/or treating PJI. Future studies should focus on optimisation of immunomodulator-based approaches such as fMLP dose assessment, implant coating with fMLP or combination of fMLP with prophylactic antibiotics or other immunomodulators.

## Supplementary Material

Video

## List of Abbreviations

AMP: antimicrobial peptide
ANOVA: analysis of variance
BLI: bioluminescence imaging
CFU: colony-forming unit
CI: confidence interval
DMSO: dimethyl sulphoxide
EDTA: ethylenediaminetetraacetic acid
fMLF: *N*-formyl-methionyl-leucyl-phenylalanine
fMLP: *N*-formyl-methionyl-leucyl-phenylalanine
FPR: formyl peptide receptor
H&E: haematoxylin and eosin
ICC: intraclass correlation
K-wire: Kirschner wire
MPO: myeloperoxidase
NETs: neutrophil extracellular traps
NIH: National Institutes of Health
PBS: phosphate-buffered saline
PJI: periprosthetic joint infection
PRRs: pattern recognition receptors
PWT: paw withdrawal threshold
ROI: region of interest
ROS: reactive oxygen species
*S. aureus*: *Staphylococcus aureus*
SEM: standard error of the mean
SSI: surgical site infection
TSB: tryptic soy broth
μCT: micro-computed tomography
